# Circulating Interleukins and Risk of Multiple Sclerosis: A Mendelian Randomization Study

**DOI:** 10.3389/fimmu.2021.647588

**Published:** 2021-04-15

**Authors:** Hui Lu, Peng-Fei Wu, Wan Zhang, Xiaoyao Liao

**Affiliations:** ^1^ Department of Neurology, Xuanwu Hospital, Capital Medical University, Beijing, China; ^2^ Hunan Provincial Key Laboratory of Medical Genetics, School of Life Sciences, Central South University, Changsha, China; ^3^ Department of Neurology, Beth Israel Deaconess Medical Center and Harvard Medical School, Boston, MA, United States; ^4^ Department of Biology, College of Arts & Sciences, Boston University, Boston, MA, United States; ^5^ College of Medical, Veterinary and Life Sciences, University of Glasgow, Glasgow, United Kingdom

**Keywords:** interleukins, multiple sclerosis, Mendelian randomization, genome wide association study, single-nucleotide polymorphisms

## Abstract

**Background:**

Previous research have implicated critical roles of systemic inflammation in the development of Multiple Sclerosis (MS). But the causal relationship between interleukins (ILs) and MS has not been fully elucidated.

**Objective:**

In this study, we applied Mendelian randomization (MR) approaches to address the causal associations between genetically determined circulating levels of ILs and the risk of MS.

**Methods:**

Genetic instruments for circulating IL-1 receptor antagonist (IL-1Ra), IL-2 receptor α subunit (IL-2Rα), IL-6, IL-16, IL-17, and IL-18 were obtained from recently published genome-wide association studies (GWAS). Summary-level data for MS were obtained from the International Multiple Sclerosis Genetics Consortium. MR analyses were performed using the R software (version 3.6.1, The R Foundation) and the TwoSampleMR package.

**Results:**

Genetic predisposition to higher circulating levels of IL-2Rα were significantly associated with MS risk. The odds ratio (OR) was 1.22 (95% confidence interval [CI], 1.12–1.32; *p* < 0.001) per one standard deviation increase in circulating IL-2Rα levels. There was a suggestive association of circulating IL-1Ra with MS risk (OR, 0.94; 95% CI, 0.88–0.99; *p* = 0.027). The other ILs were not associated with the outcome.

**Conclusion:**

Our results indicated that circulating IL-2Rα was causally associated with risk of MS.

## Introduction

Multiple sclerosis (MS) is the most prevalent chronic inflammatory disease of the central nervous system (CNS), affecting more than 2 million people worldwide (1). It is the leading non-traumatic cause of disability in young individuals, characterized most commonly by impaired ambulation, slowed cognitive processing, and/or loss of bladder control (2, 3). Currently, no medication can fully prevent or reverse the progressive neurological deterioration of MS (3). MS costs approximately 10 billion dollars annually in the United States (4). The definite etiology of MS remains unclear. The chronic course of MS makes it susceptible to multiple interacting risk factors, including genetic polymorphisms and environmental exposures (5). Numerous studies have implicated that systemic inflammation contributes to MS etiopathogenesis and also serves as targets for MS treatments (6). Furthermore, most risk alleles of MS identified by genome-wide association study (GWAS) are related to immune-pathway genes (3).

Data addressing MS pathogenesis were chiefly gained from an animal model of MS, the experimental autoimmune encephalomyelitis (EAE). The inflammatory process in EAE is initiated by the binding of pathogen-associated molecular patterns or damage-associated molecular patterns to pathogen recognition receptors, resulting in the activation of innate immune cells and the production of interleukin (IL)-1, IL-6, IL-12, IL-18, and IL-23 which promote the differentiation and expansion of T helper (Th) 1 and Th17 cells (7). IL-1β and IL-23 activate γδ T cells, and γδ T cells further secrete IL-17 & IL-21 (6). Activated Th1, Th17, and γδ T cross the blood-brain barrier and secrete IL-17, granulocyte and macrophage colony stimulation factor (GM-CSF), interferon gamma (IFN-γ), and tumor necrosis factor (TNF) (6). IL-2 is mainly expressed by activated T cells and has paradoxical roles in both immunity and the maintenance of T-cell tolerance (8). IL-2 makes its activity by specifically binding to IL-2 receptor (IL2R) (9). IL2R is expressed on the surface of various immune cells, varying from antigen-presenting cells, to conventional T cells and T regulatory cells (Treg) (10). The IL-2 signaling plays a critical role in the competitive-fitness maintenance of the peripheral Treg cells and the inhibition of the Th17 cells response (10). IL-2 receptor α subunit (IL-2Rα), also known as CD25, has the highest affinity among the three subunits of IL2R (11). IL-16 is an important regulator of CXCR4 signaling and IL-2Rα expression (12).

However, the potential causative role of individual cytokines on MS risk in human beings remains elusive. So it is imperative to develop valid strategies to identify cytokines that are causally linked to the risk of MS.

Leveraging germline genetic variants as proxies, Mendelian randomization (MR) approach is used to evaluate the causal role of exposures on outcomes (13). Since the random assignment of genetic variants happens at gametogenesis and is accordingly unaffected by environmental factors or disease process, MR diminishes confounding (14), strengthens the exposure-outcome association and avoids reverse causality (13, 15). With MR analysis, body mass index and serum 25-hydroxyvitamin D have been confirmed to be casually linked to the development of MS (16). But only a few MR studies addressed the causal relationship between circulating cytokines and MS. One previous MR study has verified significant association of genetically increased serum IL-6 receptor (IL-6R) levels with reduced risk of MS, and speculated that inhibiting IL-6 signaling (such as tocilizumab treatment) would be an effective therapy for MS (17). While an atlas on risk factors for MS did not found causal role of TNF on the risk of MS (16).

Among inflammatory components, ILs are a large group of cytokines secreted by cells and bind to specific receptors (18). ILs play a pivotal role in triggering and modulating the immune responses in MS (19). GWAS data sets have identified one or more single nucleotide polymorphisms (SNPs) for other widely-reported circulating ILs in MS, such as IL-1 receptor antagonist (IL-1Ra), IL-2Rα, IL-6, IL-16, IL-17, and IL-18 (20–24). However, their relationship with MS has not been fully exploited by MR approach.

Here, by leveraging data from the largest GWAS to date on circulating levels of ILs, we implemented a two-sample MR analysis to appraise the associations between genetic predispositions to these circulating ILs and the risk of MS.

## Materials and Methods

This MR study is based on publicly shared databases and no additional participant ethical consent is required.

**Figure 1:**
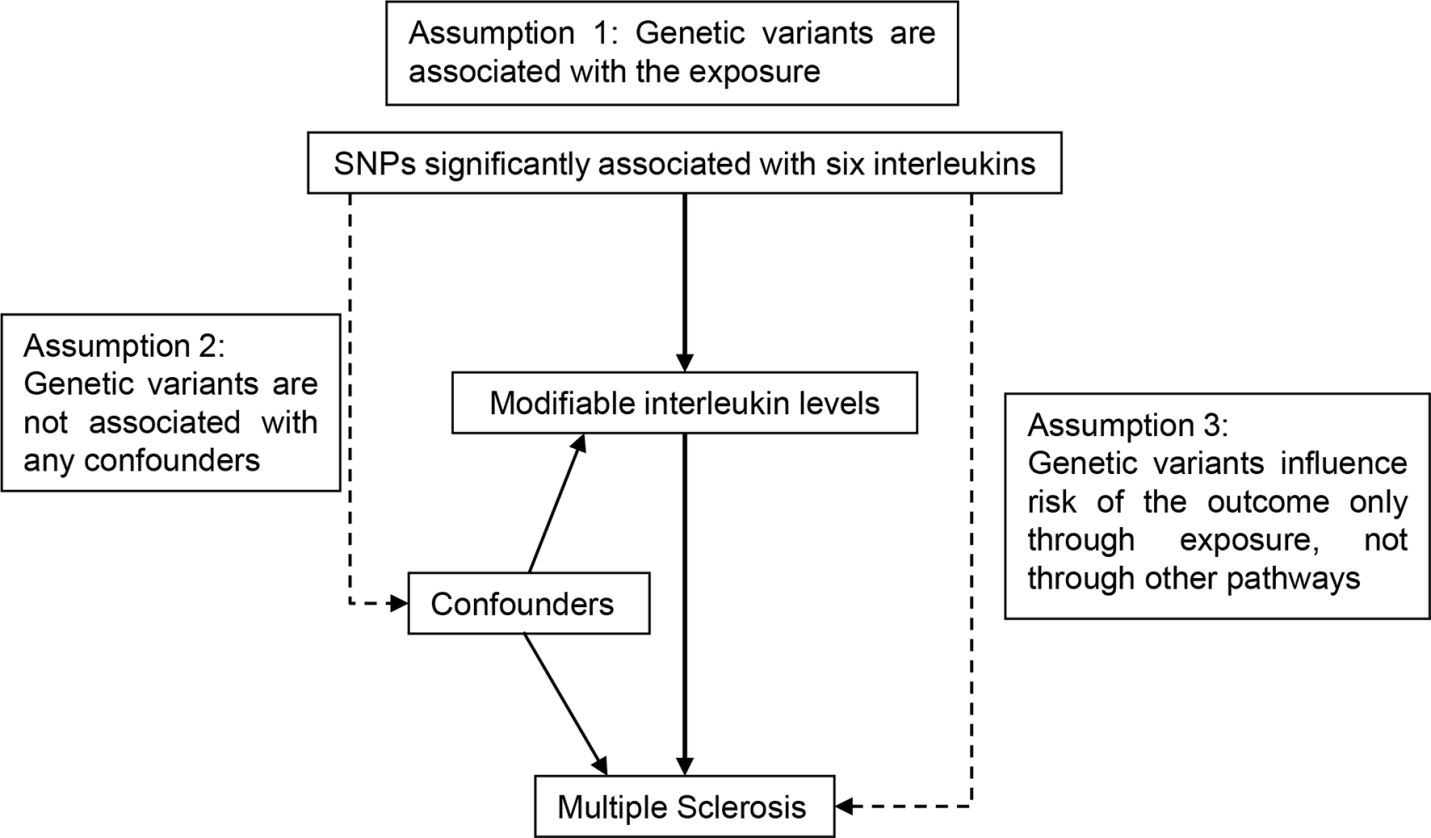
Schematic diagram for the Mendelian randomization study exploring causal effects of interleukins on the risk of multiple sclerosis. SNP, single nucleotide polymorphism.

The concept of the MR design is demonstrated in **Figure 1**. We selected instrumental SNPs associated with circulating ILs (**Table 1**, **Supplemental Table 1**) from previously published European-ancestry GWAS (20–24). First, relevance assumption was met considering that all SNPs have reached genome-wide significance (*p* < 5 × 10^−8^). Second, independence assumption was validated given that the assortment of alleles is earlier than the onset of diseases, which is a natural randomization by Mendel’s law and not prone to such confounding factors as social-economic status in observational studies. Third, since instrumental SNPs associated with ILs were mainly mapped to the loci where IL-coding genes located, their strong biological implications preclude alternate pathways. Summary-level data for MS was retrieved from the shared data set by the International Multiple Sclerosis Genetics Consortium (25). From the European population, 14,802 MS cases and 26,703 healthy participants were included. Demographic information of contributing cohorts, like age, gender and diagnosis criteria, was available in the original GWAS. The data set can be accessed upon request (http://imsgc.net/).

**Table 1:** Single-nucleotide polymorphisms selected as instrumental variables associated with circulating interleukins and their associations with multiple sclerosis.

Interleukins	SNP	Nearest gene	Chr	EA	EAF	Interleukins			Multiple sclerosis		
						Beta	SE	*P*-value	Beta	SE	*P-*value
IL-1Ra	rs1542176	RNU6-1180P	2	C	0.51	0.33	0.05	2.1 × 10^−12^	−0.01	0.02	0.44
IL-1Ra	rs6743376	IL1F10	2	C	0.31	0.46	0.05	2.4 × 10^−22^	−0.04	0.02	0.03
sIL-2Rα	rs12722497	IL2RA	10	A	0.14	0.63	0.05	1.6 × 10^−38^	0.12	0.03	3.2 × 10^−6^
IL-6	rs643434	ABO	9	G	0.62	−0.26	0.02	1.4 × 10^−48^	0.03	0.02	0.06
IL-6	rs56383622	IL6R	1	A	0.61	−0.23	0.02	1.3 × 10^−22^	0.03	0.02	0.05
IL-16	rs4778636	IL16	15	A	0.04	−0.73	0.06	1.1 × 10^−30^	0.03	0.03	0.36
IL-17	rs1530455	PDIA5	3	C	0.64	−0.11	0.02	4.7 × 10^−10^	−0.03	0.02	0.10
IL-18	rs2250417	BCO2	11	T	0.48	0.10	0.01	1.9 × 10^−32^	−0.02	0.02	0.32
IL-18	rs7577696	DPY30/SPAST	2	G	0.41	0.08	0.01	2.7 × 10^−19^	−0.01	0.02	0.46

SNP, single-nucleotide polymorphism; Chr, chromosome; EA, effect allele; EAF, effect allele frequency; SE, standard error, IL-1Ra, interleukin-1 receptor antagonist; sIL-2Rα, soluble IL-2 receptor α subunit.

We conducted a two-sample MR using the R programming language (Version 3.6.1;R Foundation for Statistical Computing, Vienna, Austria), with the TwoSampleMR package (Version 0.5.3) (9). First, the SNP-exposure association data set was curated after checking for linkage disequilibrium (threshold set at *R^2^* > 0.01, within 1 Mb window, the EUR panel of 1000 Genomes Project Phase 3). The SNP with lowest *P*-value at each locus was kept as instrumental variable. Second, corresponding statistics for the SNP-outcome association were retrieved from the whole MS data set containing 8,589,719 SNP records. As for SNPs not available in the original outcome data set, SNPs reaching minimum linkage disequilibrium threshold (*R^2^* > 0.8) were used as proxies. Third, summary-level statistics of interleukin and MS were harmonized to make sure the effect of a given SNP on the exposure and on the risk of MS corresponds to the same effect allele. Palindromic alleles (A/T, or G/C) with intermediate minor allele frequency (MAF > 0.45) were dropped since the aligned strand might be ambiguous and not inferable.

Using the harmonized data set, MR analyses were performed and related graphs were produced. First, the Wald ratio for each instrumental variable were derived by dividing the SNP-outcome effect size by the corresponding SNP-exposure coefficient. For each instrument, the SNP-exposure effect size *X_k_* with the standard error $\sigma_{X_{k}}$, and the SNP-outcome association *Y_k_* and $\sigma_{Y_{k}}$, the MR estimate can be given by the Wald ratio *Yk*/*Xk* with the standard error $\sigma_{Y_{k}}X_{k}$. Then the inverse-variance weighted gave an overall causal estimator ${\hat{\beta }}_{MR}$ and ${\hat{\sigma }}_{MR}$ its standard error by the following formula.

$${\hat{\beta }}_{MR}=\sum X_{k}Y_{k}\sigma_{Y_{k}}^{-2}/\sum X_{k}^{2}\sigma_{Y_{k}}^{-2}$$

$${\hat{\sigma }}_{MR}=1/\sum X_{k}^{2}\sigma_{Y_{k}}^{-2}$$

Funnel plots and scatter plots were made to show the overall causal estimate and individual effect of each SNP. The odds ratio (OR) and 95% confidence interval (CI) represented the risk for MS per one standard deviation increase in circulating concentrations of ILs. Statistical significance was set at *p* < 0.05/6 using the Bonferroni correction.

## Results

Summary statistics of instrumental SNPs as genetic instrumental variables for six types of circulating ILs were presented in **Table 1**. Corresponding associations with MS were extracted. Notably, one SNP mapped at sIL-2Ra, rs12722497 was associated with MS (*p* = 3.2 × 10^−6^) at suggestive genome-wide significance. Other instrumental SNPs showed no significant association with the susceptibility to MS.

**Figure 2:**
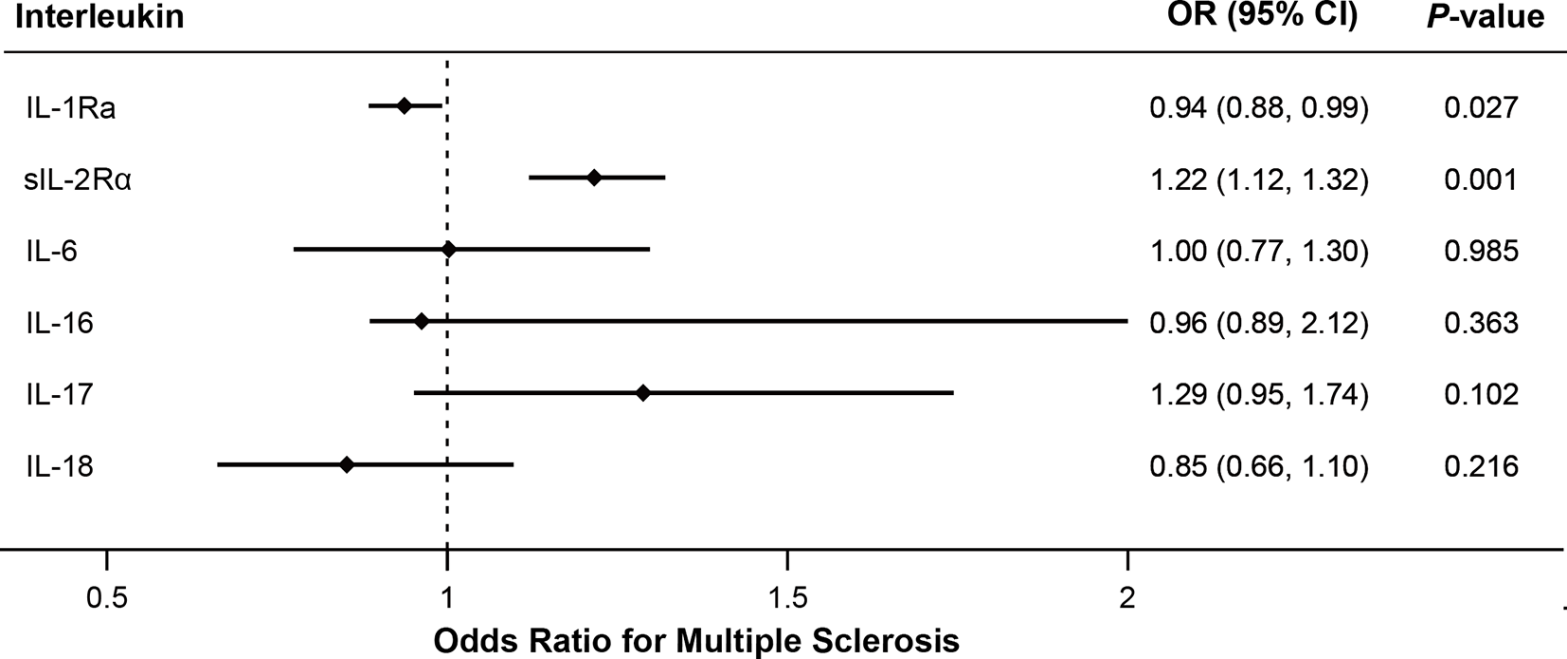
Mendelian randomization results for the relationship between interleukins and multiple sclerosis. IL-1Ra, interleukin-1 receptor antagonist; IL-2Rα, IL-2 receptor α subunit; OR, odds ratio; CI, confidence interval.

**Figure 3:**
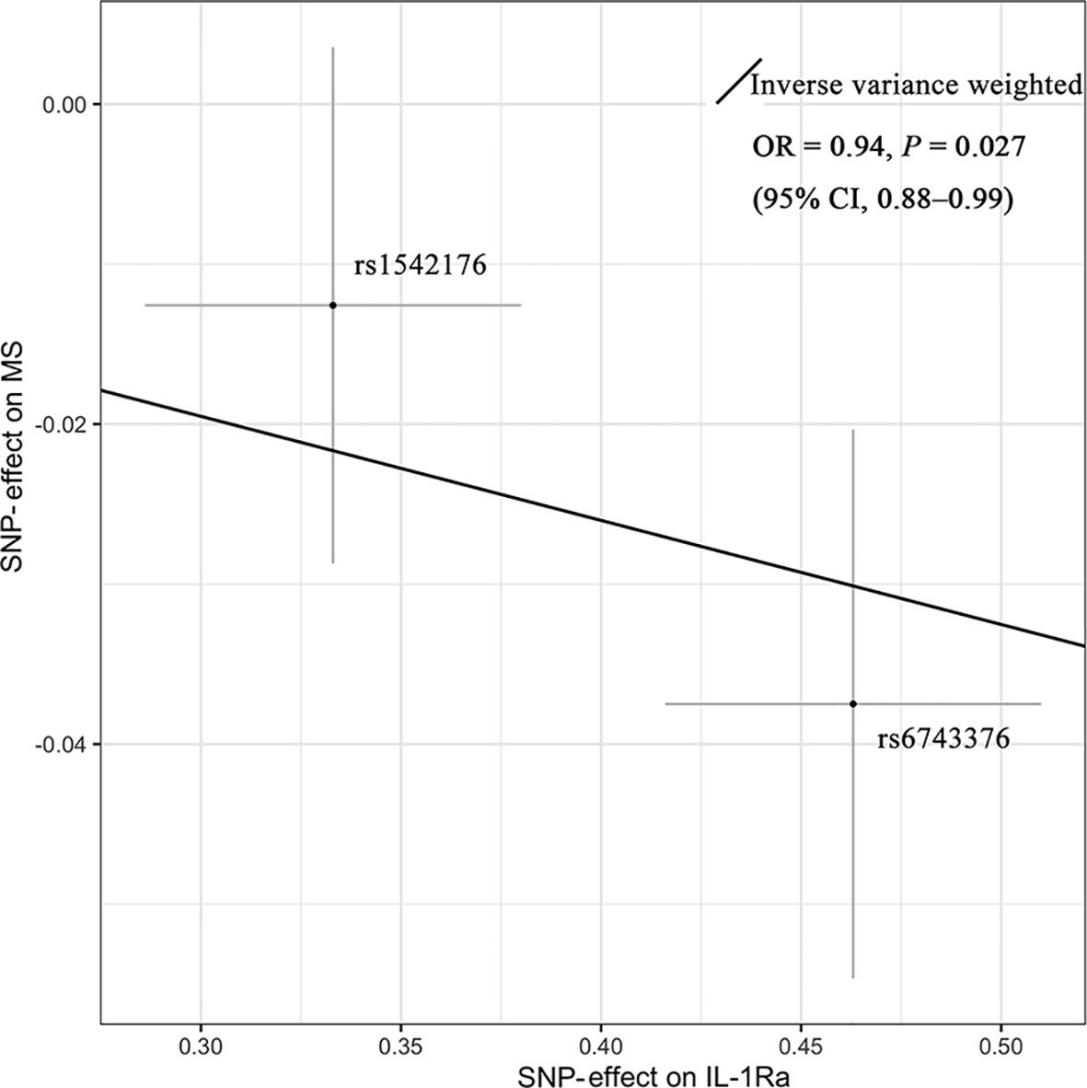
Mendelian randomization scatter plot showing the effect of IL-1Ra on MS. There was suggestive evidence (*p* = 0.027) supporting the association between elevated concentrations of IL-1Ra and the lower risk of MS (OR = 0.94; 95% CI, 0.88–0.99). The causal estimate by the inverse-variance-weighted method was presented as the overall fitted line. Individual SNP-effect on the risk of MS (point and vertical line) against its effect on the IL-1Ra (point and horizontal line) was delineated in the background. CI, confidence interval; IL-1Ra, interleukin-1 receptor antagonist; MS, multiple sclerosis; OR, odds ratio; SNP, single-nucleotide polymorphism.

**Figure 4:**
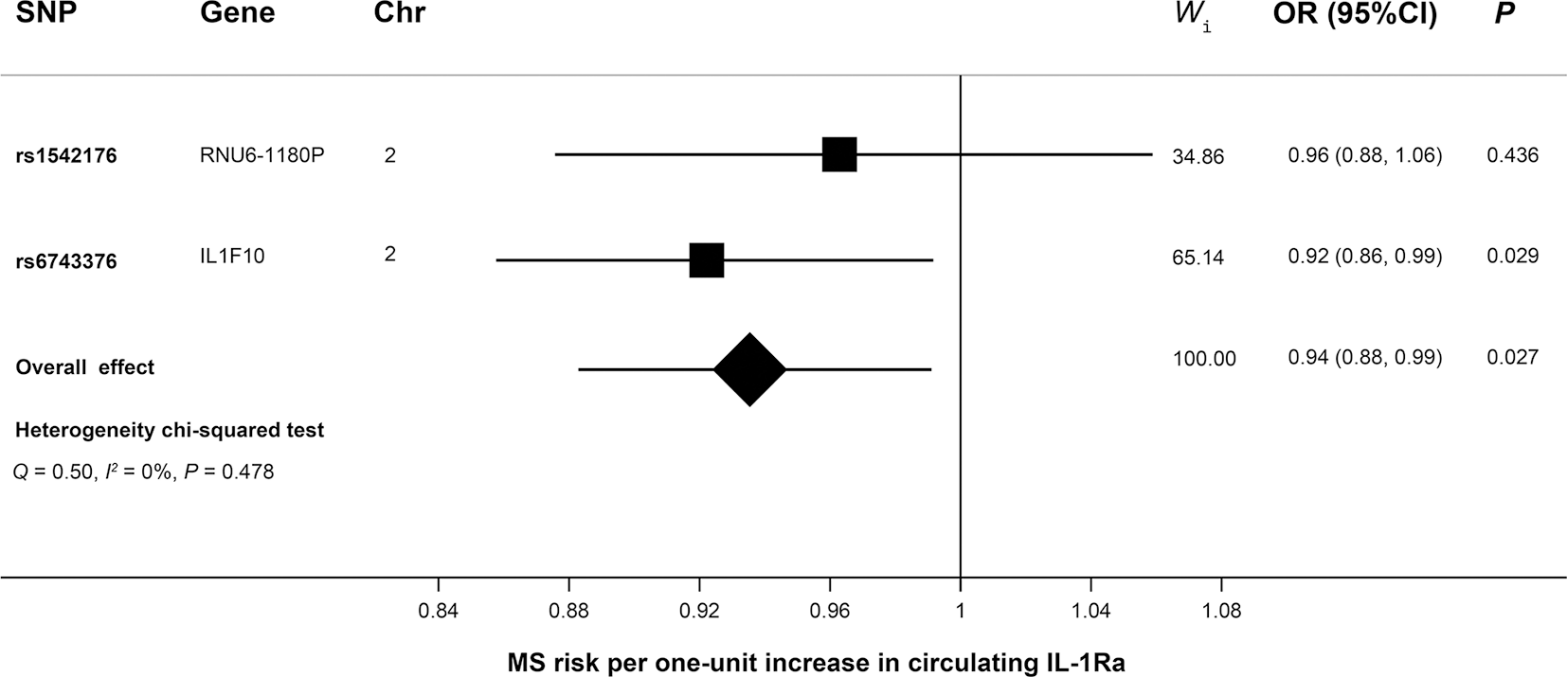
Mendelian randomization forest plot showing the effect of IL-1Ra on MS. Causal estimate of individual SNP was presented using the square box, while the overall estimate was illustrated using the diamond box. Chr, chromosome; CI, confidence interval; IL-1Ra, interleukin-1 receptor antagonist; MS, multiple sclerosis; OR, odds ratio; SNP, single-nucleotide polymorphism; Wi, weight of corresponding SNP in the inverse variance-weighted MR.

As shown in **Figure 2**, MR analyses suggested genetically predicted higher circulating levels of sIL-2Rα were significantly associated with increased risk for MS (OR, 1.22; 95% CI, 1.12–1.32; *p*<0.001). There was suggestive evidence for the association between circulating IL-1Rα and MS risk (OR, 0.94; 95% CI, 0.88–0.99; *p* = 0.027). Notably, the effect seems to be partly driven by rs6743376 which located at *IL1F10* (**Figures 3** and **4**); nevertheless, there was no evidence of heterogeneity (*Q* = 0.50, *p* = 0.478). Overall MR results did not support the role of IL-6 (OR, 1.00; 95% CI, 0.77–1.30; *p* = 0.985) in the risk of MS (**Supplemental Figures 1** and **2**). Considering the potential pleiotropic effect of rs643434 which located at the complex ABO region, the relationship between IL-6 and MS risk was also estimated using the single SNP rs56383622 located at *IL6R*; there was suggestive evidence demonstrating that elevated levels of IL-6 were associated with lower risk of MS (OR, 0.87; 95% CI, 0.75–1.00; *p* =0.047). MR results (**Supplemental Figures 3** and **4**) did not support the causal effect of IL-18 on MS risk (OR, 0.85; 95% CI, 0.66–1.10; *p* = 0.216), and there was no significant heterogeneity (*Q* =0.01, *p* =0.976). There was no evidence for the association between IL-16 (OR, 0.96; 95% CI, 0.89–2.12; *p* = 0.363) and IL-17 (OR, 1.29; 95% CI, 0.95–1.74; *p* = 0.102) and the predisposition to MS.

## Discussion

We identified genetic predisposition to higher levels of circulating IL-2Rα is associated with higher risk of MS. We also found weak evidence for circulating IL-1Ra in determining the risk of MS. Although after correction for multiple comparisons, it did not reach statistical significance. The other assessed circulating ILs (IL-6, IL-16, IL-17, and IL18) were not associated with MS risk.

Notably, rs12722497 is mapped to the 1st intro of the protein-coding gene *IL2RA*, located at 10p15.1. Albeit as an intronic variant, it has also been identified as an expression quantitative trait locus for *IL2RA*, which is constitutively expressed on regulatory T cells and plays an essential role in the immune system, such as tolerance regulation and T-cell expansion. Besides, rs12722497 is a protein quantitative trait locus for circulating IL-2Rα. The circulating IL-2Rα, namely, soluble IL-2Rα (sIL-2Rα), is the shedding of the IL-2Rα chain from the surface of activated T cells. sIL-2Rα significantly causes exacerbation of disease symptoms in EAE (26). The causal association in our study between genetically determined levels of circulating IL-2Rα and MS risk is consistent with that. The function of sIL-2Rα in IL-2 signaling is controversial, with both antagonistic and agonistic roles. It may either act as a decoy-receptor for IL-2 or it enables IL-2 signaling by trans presentation of IL-2 to IL-2R expressing T cells (10). In follicular B-cell non-Hodgkin lymphoma, sIL-2Rα enhances STAT5 phosphorylation mediated by IL-2, up-regulates Foxp3 expression in CD4+ T cells, and promotes T-cell differentiation to Treg rather than Th1 or Th17 (27). While in EAE, sIL-2Rα inhibits STAT5 phosphorylation in both conventional T-cells and Treg (28). The pathogenicity of sIL-2Rα in EAE is associated with enhanced generation of Th17 in the periphery and increased infiltration of both Th1 and Th17 cells into CNS (26). The conflicting results in these two diseases might be explained by discrepant ratios of sIL-2Rα versus IL-2 (27). Daclizumab, a humanized monoclonal antibody against IL-2Rα, inhibits IL-2 binding to IL-2R and also blocks sIL-2Rα binding to IL-2 (8). Phase II and III clinical trials has proved that regular injection of Daclizumab can decrease the number of gadolinium-enhanced lesions on brain MRI and significantly reduce the annualized relapse rate of MS (29). To the best of our knowledge, this is the first MR study to show a robust causal association between circulating IL-2Rα and MS risk. This study strengthens the evidence that targeting circulating IL-2Rα may offer a therapeutic approach for MS.

Another SNP of particular interest was rs6743376, mapped to the Extron 4 of *IL1F10* (interleukin 1 family, member 10) at 2q14.1. It is a missense variant with the amino acid changing from alanine to aspartic acid, which is likely to alter the protein property and effectiveness. Furthermore, rs6743376 is a protein quantitative trait locus for IL-1Ra. The IL-1 family includes two agonist (IL-1α and IL-1β) and an antagonist protein (IL-1Ra) (30). IL-1β initiates the demyelination process of MS and has a direct neurodegenerative effect (31, 32). The presence of IL-1β in white matter or acute lesions has been found to be correlated with cortical lesion load (33, 34). IL-1Ra competes with IL-1α or IL-1β for the IL-1 receptor and inhibits the activation of pathogenic Th17 cells in MS (30). IL-1Ra has been proposed to have anti-inflammatory activity and down-regulate the pro-inflammatory effect of IL-1β (35). IL-1RA along with CSF-1, CSF-2, and IL-17 are related to the disturbance of IL-1 signaling which leads to immune activation with a shift towards autoimmune Th2 response in the CNS (36). Recently, dysregulated levels of IL-1Ra has been indicated in MS (37). Elevated IL-1β/IL-1Ra ratio in cerebrospinal fluid and blood of MS patients has a correlation with disease susceptibility, severity, and progression (34, 38–42). Currently, no anti–IL-1 drugs has been routinely used in MS due to paradoxical effects of IL-1 that has strong proinflammatory function and also neuroprotective & tissue-remodeling function (43). However, immunomodulators (i.e. interferon-β, glatiramer acetate, natalizumab, or steroid) that increase circulating IL-1Ra levels and cause remission of disease in MS patients, are widely used in MS therapy (44). Our results of this study implicated a weak inverse correlation between levels of IL-1Ra and MS risk, and further investigations are warranted to find out whether it can be a therapeutic targets for MS treatment.

There are several strengths in this study. First, we employ MR analysis to minimize confounding and to avoid reverse causality. Second, we used the most recent data set for interleukins and the largest GWAS data set for MS to systematically evaluate their causal relationship. Third, leveraging summary-level data set of large-scale genetic consortia, we had high statistical power to detect weak associations of circulating ILs with MS. Limitations also exist in our study. First, non-linear effect of circulating ILs levels on risk of MS, such as a U-shaped association or a threshold effect, cannot be examined. Second, our database originated predominantly from European-ancestry studies and this restriction reduced the transferability to other ethnicities worldwide. Third, the restriction of our study population to European ancestry hampered the generalization to individuals of other ancestries. Lastly, we only analyzed limited types of ILs, and further studies are warranted to investigate the role of other ILs (such as IL-12, IL-21, IL-23, etc) for the risk of MS.

## Conclusion

Collectively, this MR study suggests that elevated levels of circulating IL-2Rα increase the risk of MS. Our result provides the genetic support that targeting circulating IL-2Rα or its downstream effectors might be a meaningful strategy for the treatment of MS.

## Data Availability Statement

The original contributions presented in the study are included in the article/**Supplementary Material**. Further inquiries can be directed to the corresponding author.

## Author Contributions

HL: study design, literature research, data acquisition and manuscript preparation. P-FW: data analysis and statistical analysis. WZ: manuscript editing and manuscript revision. XL: manuscript editing and manuscript revision. All authors contributed to the article and approved the submitted version.

## Funding

This work was supported by scientific research and cultivation plan of Beijing Municipal Hospital (grant PX2021036).

## Conflict of Interest

The authors declare that the research was conducted in the absence of any commercial or financial relationships that could be construed as a potential conflict of interest.

The reviewer AB declared a shared affiliation with one of the authors, P-FW, to the handling editor.
