# Supplementary material for: Circulating Interleukins and Risk of Multiple Sclerosis: A Mendelian Randomization Study
Source: Front Immunol. 2021 Apr 15;12:647588. doi: 10.3389/fimmu.2021.647588 (PMC8081970; doi:10.3389/fimmu.2021.647588)
Supplement: Supplementary file 1 [file DataSheet_1.pdf]

Supplementary Content

Supplemental Table 1. Basic information for summary-level datasets utilized in the Mendelian randomization study **2**

Supplemental Figure S1. Mendelian randomization scatter plot showing the effect of IL-6 on MS.. **3**

Supplemental Figure S2. Mendelian randomization forest plot showing the effect of IL-6 on MS **4**

Supplemental Figure S3. Mendelian randomization scatter plot showing the effect of IL-18 on MS. **5**

Supplemental Figure S4. Mendelian randomization forest plot showing the effect of IL-18 on MS.. **6**

Supplemental Table 1. Basic information for summary-level datasets utilized in the Mendelian randomization study.

Phenotype	Author and Year	Sample Size (Cases/Controls)	Female (%)	Mean Age at Examination	Population	Main Cohort	PubMed ID
IL-1Ra	Herder et al., 2014	16,160	47.9%	51.3	European	Whitehall II	24969107
sIL-2Rα	Ahola-Olli et al., 2017	8,293	47.4%	45.3	European	FINRISK	27989323
IL-6	Russell et al., 2020	8,296	42.6%	43.6	European	SardiNIA	32473944
IL-16	Ahola-Olli et al., 2017	8,293	47.4%	45.3	European	FINRISK	27989323
IL-17	Ahola-Olli et al., 2017	8,293	47.4%	45.3	European	FINRISK	27989323
IL-18	Matteini et al., 2014	6,135	43.6%	50.8	European	CHS	24182552
Multiple Sclerosis	Patsopoulos et al., 2018	14,802 / 26,703	40.8%	45.3	European	IMSGC	31604244

Abbreviations: IL, interleukin; IL-1Ra, IL-1 receptor antagonist; sIL-2Rα, soluble IL-2 receptor α subunit; FINRISK, Finnish population-based survey on chronic disease risk factors; SardiNIA, Sardinian population-based study by National Institute on Aging; CHS, Cardiovascular Health Study; IMSGC, International Multiple Sclerosis Genetics Consortium.

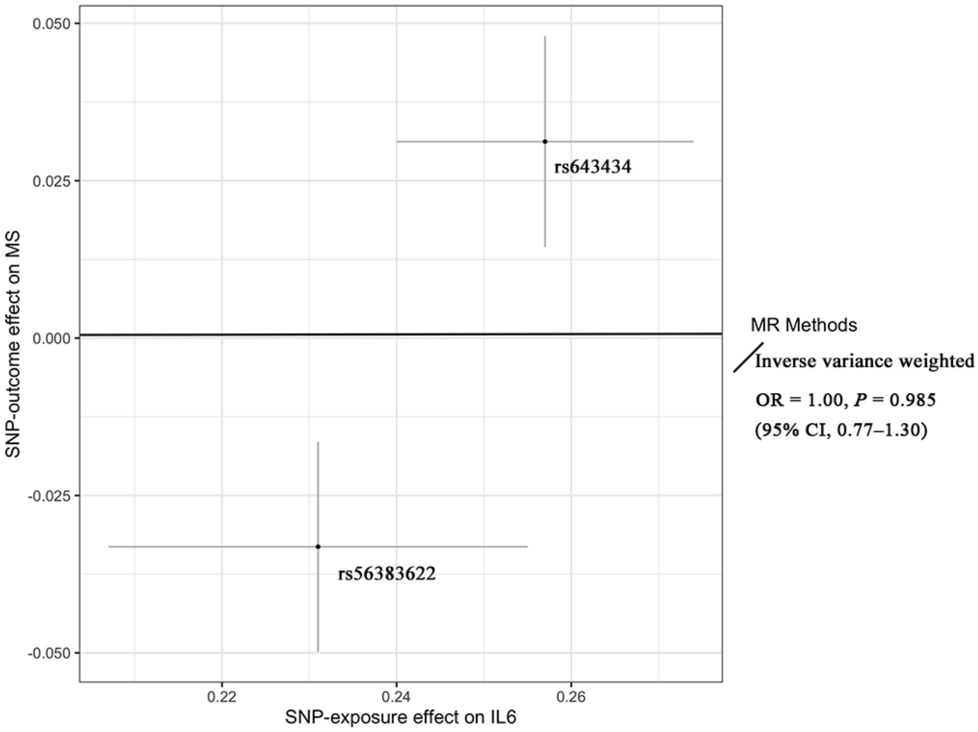

Figure S1. Mendelian randomization scatter plot showing the effect of IL-6 on MS. There was no association between circulating concentrations of IL-6 and MR risk (OR=1.00; 95% CI, 0.77-1.30; p =0.985). The causal estimate by the inverse-variance-weighted method was presented as the overall fitted line. Individual SNP-effect on the risk of MS (point and vertical line) against its effect on the IL-6 (point and horizontal line) was delineated in the background. CI, confidence interval; IL-6, interleukin-6; MS, multiple sclerosis; OR, Odds ratio; SNP, Single nucleotide polymorphism.

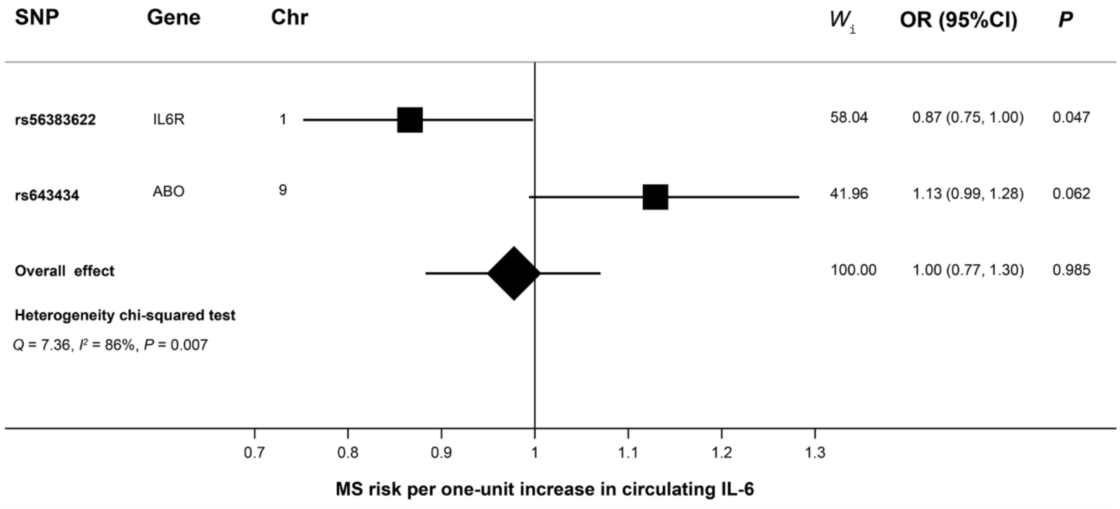

Figure S2. Mendelian randomization forest plot showing the effect of IL-6 on MS. Causal estimate of individual SNP was presented using the square box, while the overall estimate was illustrated using the diamond box. Chr, chromosome; CI, confidence interval; IL-6, interleukin-6; MS, multiple sclerosis; OR, Odds ratio; SNP, Single nucleotide polymorphism; Wi, weight of corresponding SNP in the inverse-variance-weighted MR.

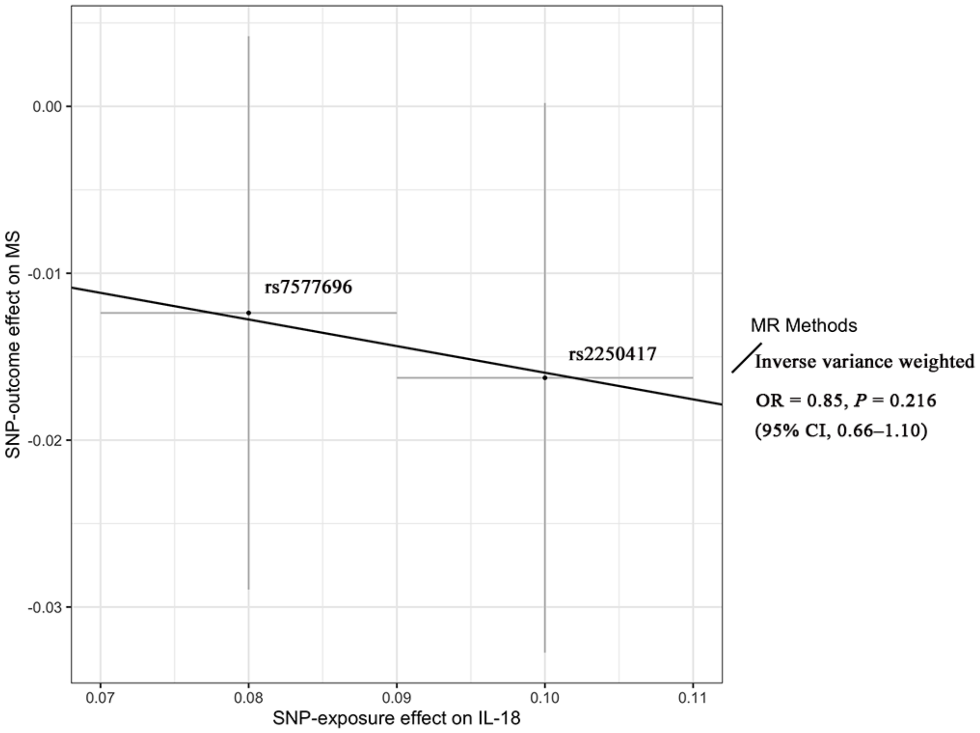

Figure S3. Mendelian randomization scatter plot showing the effect of IL-18 on MS. There was no association between circulating concentrations of IL-18 and MR risk (OR=0.85; 95% CI, 0.66-1.10; p =0.216). The causal estimate by the inverse-variance-weighted method was presented as the overall fitted line. Individual SNP-effect on the risk of MS (point and vertical line) against its effect on the IL-18 (point and horizontal line) was delineated in the background. CI, confidence interval; IL-18, interleukin-18; MS, multiple sclerosis; OR, Odds ratio; SNP, Single nucleotide polymorphism.

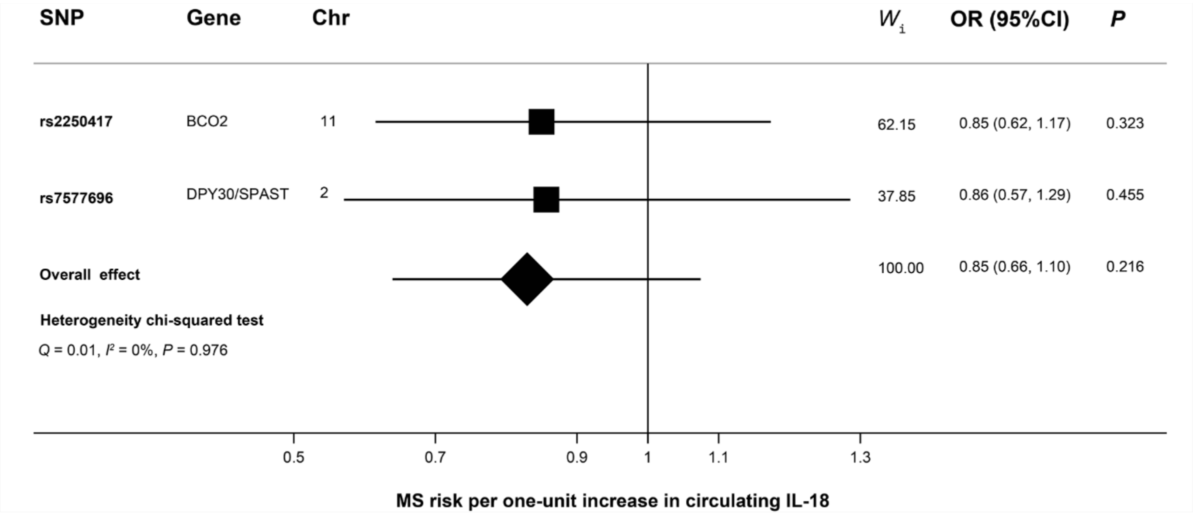

Figure S4. Mendelian randomization forest plot showing the effect of IL-18 on MS. Causal estimate of individual SNP was presented using the square box, while the overall estimate was illustrated using the diamond box. Chr, chromosome; CI, confidence interval; IL-18, interleukin-18; MS, multiple sclerosis; OR, Odds ratio; SNP, Single nucleotide polymorphism; Wi, weight of corresponding SNP in the inverse-variance-weighted MR.
